# Deciphering Drought Response Mechanisms in Oat Through Comprehensive Transcriptomic and Physiological Analysis

**DOI:** 10.3390/plants15030453

**Published:** 2026-02-01

**Authors:** Baiji Wang, Hang Yin, Xinyi Zhang, Xiangpeng Kong, Wenjie Zhao, Rui Qiu, Muzhapaer Tuluhong, Guowen Cui, Bing Li

**Affiliations:** Department of Grassland Science, College of Animal Science and Technology, Northeast Agricultural University, Harbin 150030, China; wbj011211@163.com (B.W.); yinhang361@163.com (H.Y.); zzxxyy020509@163.com (X.Z.); kl18242446011@163.com (X.K.); 13310467886@163.com (W.Z.); qr020327@163.com (R.Q.); 17645096163@163.com (M.T.)

**Keywords:** oat, drought stress, physiological indicator, transcriptomic analysis, hub gene

## Abstract

Oat, an important cereal and forage crop, is significantly affected by drought stress during production. However, the molecular mechanisms underlying oat’s response to drought stress remain largely unknown. In this study, K-means clustering classified 28 oat varieties into drought-tolerant (Muda, Mengshi No. 1) and drought-sensitive (Heike, Haywire) groups, with grey relational analysis further verifying MS as the most drought-tolerant and HK as the most drought-sensitive variety. Under drought stress, drought-tolerant and drought-sensitive varieties showed notable differences in leaf chlorophyll content, osmoregulation substances, and the activities of antioxidant enzymes. Transcriptomic analysis showed that 1915 differentially expressed genes (DEGs) were shared among all comparisons between treatment groups and the control group. KEGG pathway analysis revealed enrichment in pathways such as plant–pathogen interactions, plant hormone signal transduction, and starch and sucrose metabolism. In the signal transduction of plant hormones, eight *PP2C* genes associated with ABA signaling were increased, indicating that oats might respond to drought by enhancing metabolic activities via the ABA signaling pathway. WGCNA identified gene modules significantly associated with physiological traits. Notably, Mantel tests revealed that six core genes exhibited a positive correlation with CAT activity in the drought-tolerant variety, while showing an opposite trend in the sensitive variety. This study provides insights into the mechanisms of drought tolerance in oats and aids in the molecular breeding of drought-tolerant varieties.

## 1. Introduction

Oats (*Avena sativa* L.), an annual herbaceous plant belonging to the genus *Avena* within the family Gramineae, are extensively cultivated worldwide as a cereal feed crop due to their significant biomass, protein content, and digestible fiber [1]. In China, the arid and semi-arid regions of Shanxi, Shaanxi, Qinghai, and Gansu constitute the primary oat cultivation areas [2]. It is worth noting that in the context of global climate change [3], the phenomena of land degradation and desertification are becoming increasingly severe, particularly as the primary regions for oat cultivation are situated in arid and semi-arid zones. As a result, oats are particularly vulnerable to substantial drought stress during the growing season, leading to fluctuations in yield and a decline in quality. This vulnerability further limits the stability of oat production and its potential for industrial development.

Drought induces a series of adverse effects on plants. It not only leads to oxidative damage through the excessive accumulation of reactive oxygen species (ROS) within plant tissues [4], but also disrupts metabolic activities, including gas exchange between leaves and cells, ultimately reducing crop yields [5]. To handle drought stress, plants utilize enzymatic antioxidants like superoxide dismutase (SOD), peroxidase (POD), and catalase (CAT), along with non-enzymatic mechanisms such as glutathione, to neutralize reactive oxygen species [6,7]. Meanwhile, osmotic adjustment (OA) maintains cellular turgor pressure and normal functions by accumulating compatible solutes such as soluble sugars, proline, and organic acids [8,9,10]. Additionally, abscisic acid (ABA), as a key drought response signal, enhances plant drought resistance by regulating stomatal movement, root growth, and ABA-dependent pathways [10,11].

However, physiological studies alone are insufficient to systematically explain the complex drought response mechanisms, as the morphological changes and synthesis of these enzymes and hormones are influenced by specific metabolites and variations in gene expression. Therefore, it is crucial to elucidate the intricate drought response mechanisms through molecular techniques. Recent advancements in high-throughput technologies have propelled the rapid development of transcriptomics research [12]. Over the past few years, RNA sequencing (RNA-seq) has been extensively employed to elucidate the complexity of gene expression and regulatory networks in various plant species responding to abiotic stresses, including soybean (*Glycine max*), rice *(Oryza sativa*), alfalfa (*Medicago sativa*), and maize *(Zea mays)* [13,14,15,16]. Current research on the response of oats to drought stress predominantly concentrates on the growth, physiological, and biochemical parameters of individual varieties, alongside transcriptomic and metabolomic analyses. Nonetheless, few studies have systematically compared drought-tolerant and drought-sensitive varieties using transcriptomic analyses. Conducting this research is crucial for elucidating the differential mechanisms of drought resistance across various cultivars and for identifying the key genes involved.

Therefore, in this study, 28 oat germplasm resources were evaluated to identify drought-tolerant varieties, resulting in the identification of the drought-tolerant variety MS and the drought-sensitive variety HK. We conducted a comparative transcriptomic analysis between MS and HK, identifying several candidate genes that play crucial roles in drought stress response. The elucidation of these candidate genes and their mechanisms of action offers novel insights for the selection and breeding of drought-tolerant oat varieties, thereby contributing to enhanced oat yields in arid regions.

## 2. Results

### 2.1. Analysis of Seed Germination and Growth Indicators of Different Varieties Under Drought Stress

**Figure 1:**
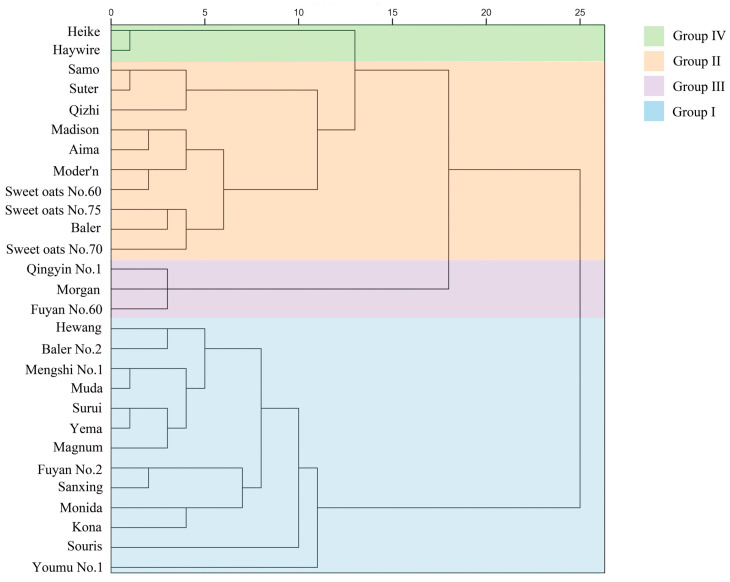
The diagram clusters oat varieties by drought resistance according to the D value, with Groups I, II, III, and IV representing various resistance levels. The D-value is the comprehensive evaluation score of drought resistance obtained for the oat varieties tested under drought stress. The values along the x-axis represent inter-cluster distances, with larger values indicating a greater degree of separation between groups.

This study first assessed the germination rate, germination potential, germination index, and the lengths of roots and shoots of 28 oat varieties under both normal conditions and drought stress induced by 25% PEG-6000. The relative values of these indicators were then calculated to perform an initial screening for drought tolerance (Supplementary Table S1). Based on the relative values of five germination indices under drought stress, a cluster analysis was performed using SPSS software (version 26.0), which organized the varieties into four categories: drought-tolerant (Group I), moderately tolerant (Group II), weakly tolerant (Group III), and sensitive (Group IV). From these groups, Muda (MD) and Mengshi No. 1 (MS) in Group I were selected as representative drought-tolerant varieties, whereas Heike (HK) and Haywire (HW) in Group IV were chosen as representative drought-sensitive varieties for subsequent experiments (Figure 1). 

### 2.2. Effects of Drought Stress on the Phenotypic and Growth Indicators of Oats

#### 2.2.1. Phenotypic Changes in Oats Under Drought Stress

**Figure 2:**
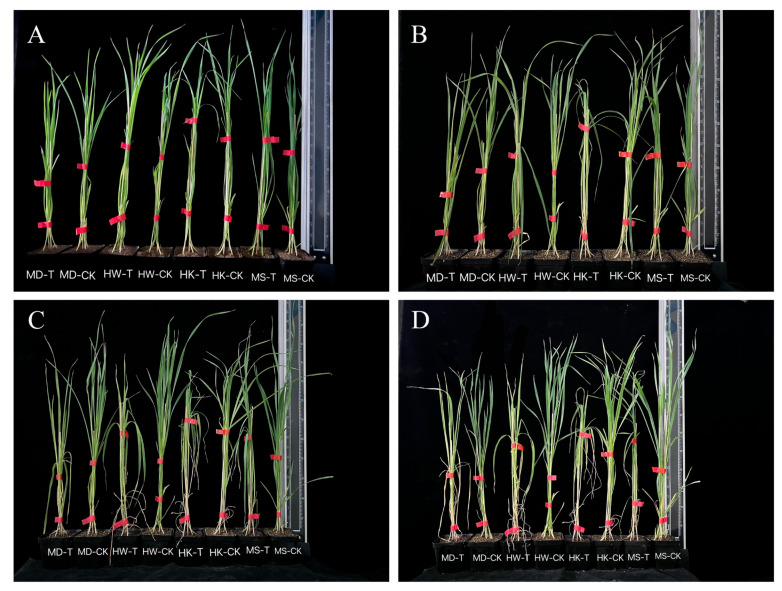
Effects of different durations of drought stress (3, 5, 7, and 10 days) on phenotypic changes (**A**–**D**).

As the drought stress prolonged, phenotypic changes in the four oat varieties were observed (Figure 2). After 5 days of stress, seedlings of HK and HW showed wilting, drooping, and yellowing of leaf tips, while MD and MS exhibited only mild leaf-tip yellowing and maintained upright growth. By day 10, all varieties experienced severe stress symptoms, including withered leaf tips and margins, along with partial plant desiccation. Notably, almost all plants of HK and HW were lodged by this stage.

#### 2.2.2. Effects of Drought Stress on Growth Indicators of Oats

**Figure 3:**
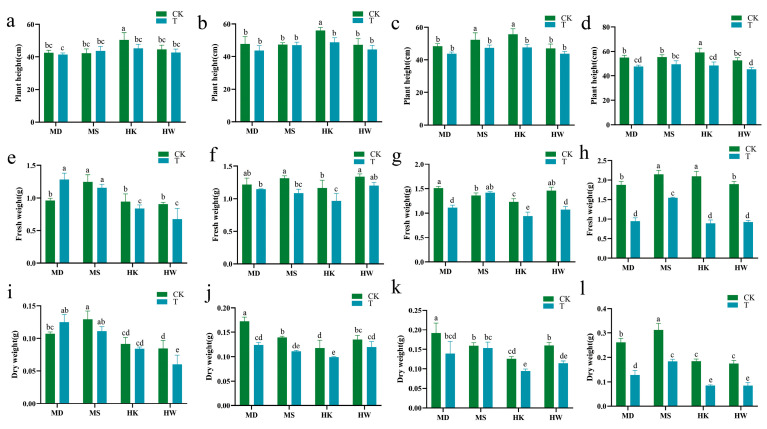
Effects of different durations of drought stress (3, 5, 7, and 10 days) on plant height (**a**–**d**), fresh weight (**e**–**h**), and dry weight (**i**–**l**) in oat. T represents the drought stress group, CK represents the control group. Different letters indicate significantly different values according to Duncan’s multiple range tests (*p*-value < 0.05).

As the duration of drought stress increased, there was a gradual reduction in the plant height of *Avena* (Figure 3a–d). By the seventh day of drought stress (Figure 3c), the plant heights of the varieties MD, MS, HK, and HW had significantly decreased by 9.5%, 9.1%, 14.7%, and 6.8%, respectively, compared to their respective controls. As shown in Figure 3e, on the third day of drought stress, the fresh weight of the drought-tolerant variety MD showed a slight increase relative to the control. However, by the tenth day (Figure 3h), the fresh weights of MD, MS, HK, and HW showed significant decreases of 49.7%, 28.0%, 57.5%, and 51.3% relative to their controls. After 10 days of drought stress, the dry weights of all four varieties exhibited a significant reduction compared to the control group. Specifically, the HK and HW varieties experienced reductions of 54.3% and 52.0%, respectively, indicating a more pronounced decline than the MD and MS varieties, which decreased by 50.8% and 41.0%, respectively (Figure 3l).

### 2.3. Effects of Drought Stress on Physiological Indicators of Oats

#### 2.3.1. Alterations in Relative Water Content

**Figure 4:**
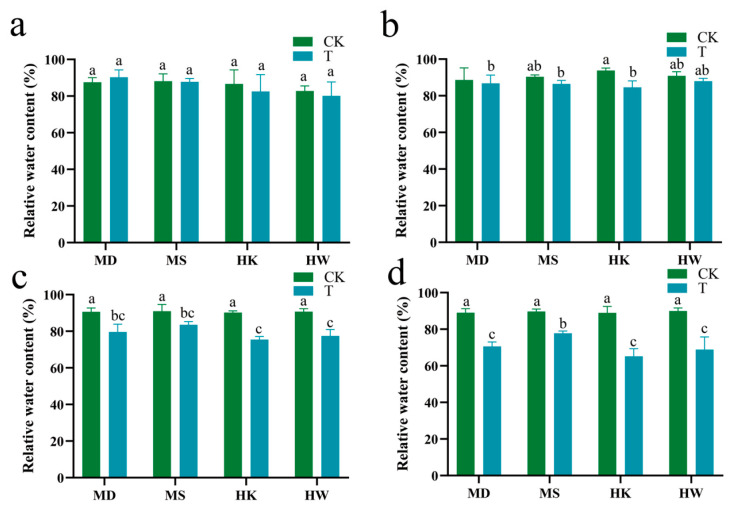
Effects of different durations of drought stress (3, 5, 7, and 10 days) on relative water content (**a**–**d**) in oat. T represents the drought stress group, CK represents the control group. Different letters indicate significantly different values according to Duncan’s multiple range tests (*p*-value < 0.05).

Under conditions of prolonged drought stress, the leaf relative water content (RWC) in oats exhibited a general declining trend (Figure 4a–d). During the early stage of drought stress, the RWC of the four cultivars showed no significant difference compared to the control group. On the seventh day, the RWC of drought-tolerant varieties MD and MS decreased significantly by 12.2% and 8.2%, respectively, compared with the control, while drought-sensitive varieties HK and HW exhibited larger reductions of 16.3% and 14.6% (Figure 4c). By the tenth day, the RWC declines in MD and MS reached 20.7% and 13.3%, respectively, whereas HK and HW showed even more significant decreases of 26.7% and 23.4% (Figure 4d). In summary, the drought-tolerant varieties demonstrated substantially superior retention of RWC compared to the drought-sensitive varieties under conditions of extended drought stress.

#### 2.3.2. Alterations in Chlorophyll Content

**Figure 5:**
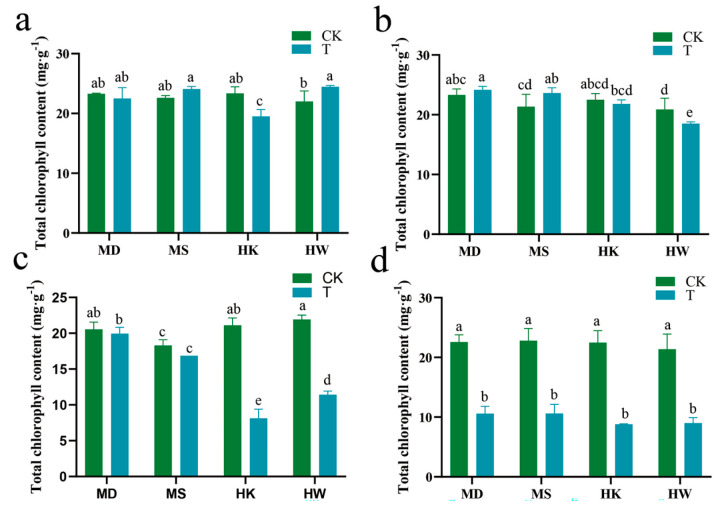
Effects of different durations of drought stress (3, 5, 7, and 10 days) on chlorophyll content (**a**–**d**) in oat. T represents the drought stress group, CK represents the control group. Different letters indicate significantly different values according to Duncan’s multiple range tests (*p*-value < 0.05).

During the progression of drought stress, chlorophyll content in oat leaves showed clear varietal differences, with drought-tolerant varieties exhibiting an overall slower declining trend (Figure 5a–d). Specifically, on the seventh day of stress, MD and MS showed only minor declines of 3% and 8%, respectively, whereas HK and HW had already exhibited sharp reductions of 58% and 48% (Figure 5c). By the tenth day of stress, chlorophyll content had decreased significantly in all varieties (Figure 5d). The drought-tolerant varieties MD and MS both experienced a reduction of 53%, while the sensitive varieties HK and HW showed even greater declines of 61% and 58%, respectively. This pattern of change indicated that drought-tolerant varieties can more effectively maintain chlorophyll stability, thereby enhancing their adaptability under drought conditions.

#### 2.3.3. Alterations in Membrane Lipid Peroxidation Markers

**Figure 6:**
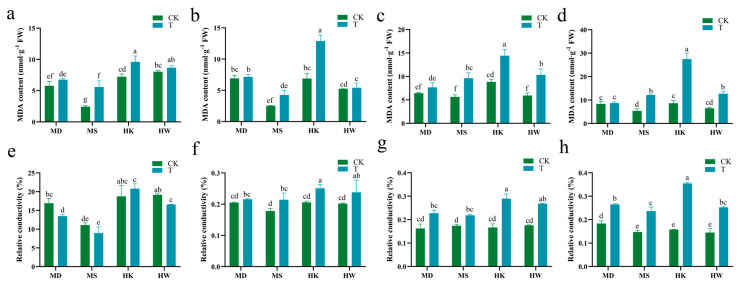
Effects of different durations of drought stress (3, 5, 7, and 10 days) on MDA content (**a**–**d**) and relative conductivity in oat (**e**–**h**). T represents the drought stress group, CK represents the control group. Different letters indicate significantly different values according to Duncan’s multiple range tests (*p*-value < 0.05).

According to Figure 6a–d, the malondialdehyde (MDA) levels in the leaves of all studied varieties increased with prolonged drought stress, but the degree of increase differed significantly. On the tenth day of drought stress, the drought-tolerant varieties MD and MS exhibited increases of 4.0% and 126.5%, respectively, relative to the control. In contrast, the drought-sensitive varieties HK and HW showed significant increases of 218.7% and 95.2%, respectively (Figure 6d). These findings suggest that membrane lipid peroxidation was least pronounced in the drought-tolerant variety MD, while the most severe peroxidation damage was observed in the sensitive variety HK.

As drought stress continued, the relative electrical conductivity (REC) of the four varieties rose gradually, with the highest values recorded on the tenth day (Figure 6h). MD, MS, HK, and HW showed significant increases of 43.2%, 61.2%, 122.9%, and 73.8%, respectively. The results demonstrated that the drought-sensitive variety HK exhibited the largest increase in leaf REC after drought stress, indicating that HK sustained the most severe cell membrane damage among the four varieties.

#### 2.3.4. Alterations in Osmotic Regulation Substances

**Figure 7:**
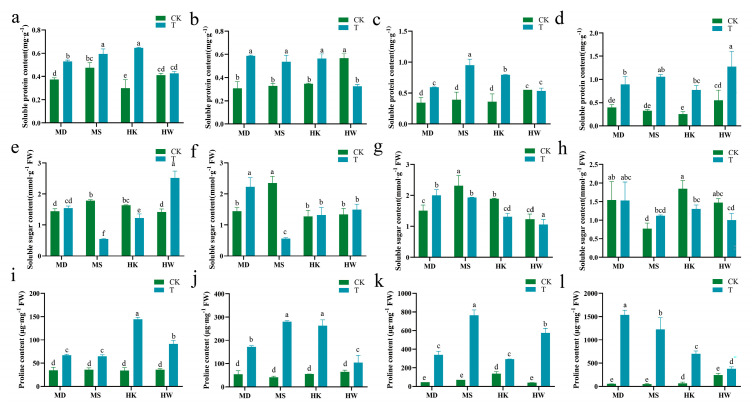
Effects of different durations of drought stress (3, 5, 7, and 10 days) on soluble protein (**a**–**d**), soluble sugar (**e**–**h**), and proline (**i**–**l**) in oat. T represents the drought stress group, CK represents the control group. Different letters indicate significantly different values according to Duncan’s multiple range tests (*p*-value < 0.05).

During extended drought stress, drought-tolerant oat varieties consistently increased soluble protein in their leaves, whereas sensitive varieties showed variable changes (Figure 7a–d). The soluble protein content in varieties MD and MS demonstrated a progressive increase, with significant elevations of 72.1% and 141.7% on day 7, and 127.1% and 223.5% on day 10 compared to the control. In contrast, varieties HK and HW displayed a fluctuating pattern characterized by an initial rise, a subsequent decline, and a final increase, peaking at day 10 with levels 203.9% and 132.1% higher than the control, respectively.

As shown in Figure 7e–h, soluble sugar content in oat leaves varied distinctly among varieties under drought stress. Specifically, the drought-tolerant variety MD continuously accumulated soluble sugars during the early stress period, reaching a peak on day 5 with a significant increase of 54.9% compared to the control, after which the levels stabilized. In contrast, the drought-tolerant variety MS exhibited a decline in soluble sugar content relative to the control in the initial phase of stress, followed by a gradual recovery, culminating in a 45.4% increase by day 10. In contrast, the sensitive varieties HK and HW exhibited greater fluctuations and inconsistent patterns in soluble sugar content, ultimately showing significant reductions of 29.3% and 31.9%, respectively, compared to the control on day 10 of stress.

Figure 7i–l depicted a consistent increase in proline content across all varieties subjected to prolonged drought stress. By day 10, the proline levels in cultivars MD, MS, HK, and HW had significantly risen to approximately 27.80, 24.89, 9.95, and 0.58 times those of the control group, respectively. The results indicated that MD and MS exhibited a more sustained and active capacity for accumulating osmotic adjustment substances, whereas the responses of HK and HW displayed greater volatility and instability.

#### 2.3.5. Alterations in Antioxidant Enzyme Activity

**Figure 8:**
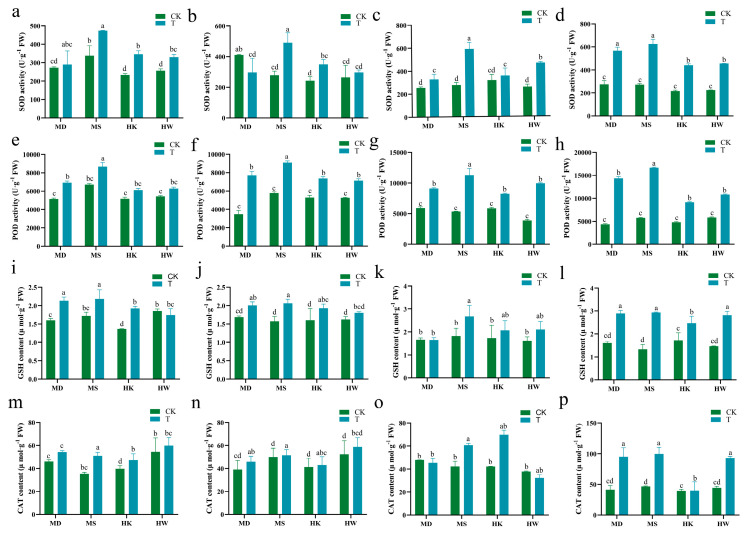
Effects of different durations of drought stress (3, 5, 7, and 10 days) on SOD (**a**–**d**), POD (**e**–**h**), GSH (**i**–**l**), and CAT (**m**–**p**) in oat. T represents the drought stress group, CK represents the control group. Different letters indicate significantly different values according to Duncan’s multiple range tests (*p*-value < 0.05).

Under prolonged drought stress, SOD activity in oat leaves demonstrated a general trend of progressive enhancement across allvarieties, culminating in peak levels by day 10 (Figure 8a–d). Specifically, the MD variety exhibited temporal variations in SOD activity, reaching its highest level on day 10 with a significant increase of 107% compared to the control. Similarly, the SOD activities of the MS, HK, and HW varieties all peaked on the tenth day of cultivation, showing significant increases of 130%, 102%, and 102%, respectively, compared to the control group.

Figure 8e–h showed that POD activity in oat leaves significantly rose under extended drought stress, peaking on day 10. Specifically, compared to the control group, the drought-tolerant varieties MD and MS showed notable increases in POD activity by 166% and 194%, respectively, while the drought-sensitive varieties HK and HW increased by 93% and 86%, respectively.

Figure 8i–l presented the alterations in GSH content in oat leaves subjected to drought stress. The GSH levels in the drought-tolerant varieties MS and MD generally showed an increase, with significant rises of 80% and 120% compared to the control group on the tenth day. The GSH content in the drought-sensitive HK and HW varieties rose significantly, by 44% and 92%, respectively, on the tenth day.

The changes in CAT activity were presented in Figure 8m–p. The varieties MD, HK, and HW exhibited a pattern of initial increase, followed by a decrease and then another rise, whereas MS showed a gradual increasing trend with prolonged drought stress duration. By the tenth day of stress, drought-tolerant varieties MD and MS increased by 165% and 113%, respectively, compared to the control. Meanwhile, drought-sensitive varieties varied: HK increased by just 1%, whereas HW surged by 109%. These results indicate that drought stress enhanced the activities of antioxidant enzymes across all four cultivars. However, among them, only the drought-tolerant variety MS exhibited consistently and significantly elevated antioxidant enzyme activity at all time points after drought stress treatment compared to the control.

### 2.4. Comprehensive Drought Resistance Evaluation Using Membership Function Analysis

This research employed a comprehensive analysis utilizing a fuzzy membership function to assess the physiological indicators of various oat cultivars subjected to drought stress. The objective was to determine the combinations exhibiting optimal drought resistance and sensitivity. Table 1 indicates that the drought resistance ranking based on D-values is: MS > MD > HW > HK, with MS being the most resistant and HK the most sensitive.

### 2.5. RNA-Seq Analysis of Oat Leaves Under Drought Stress

#### 2.5.1. Transcriptome Quality Control, Principal Component Analysis

Due to the notable variations in physiological indicators between the two oat varieties, MS and HK, a comparative transcriptome analysis was performed to explore the molecular mechanisms underlying their drought-related differences. Substantial raw data were produced from the cDNA libraries. According to Supplementary Table S2, a total of 69.03 Gb of data was filtered and obtained. For each sample, a minimum of 4.98 Gb of clean data was generated, with 84.72% to 94.11% of reads successfully mapped to the reference genome. At least 96.00% of the data achieved a Q30 quality score, and the GC content ranged from 53.00% to 54.5%.

**Figure 9:**
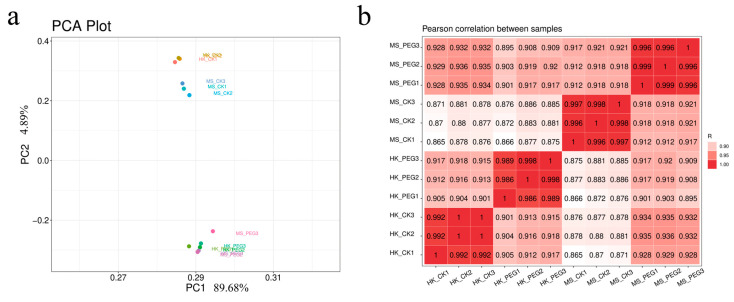
The repeatability and reliability test of the results of two varieties and their replicates. (**a**) Principal component analysis (PCA) of the similarities and differences between the 12 samples used for RNA sequencing. (**b**) Pearson’s correlation coefficient analysis of biological replicates from two oat varieties under drought stress.

Principal component analysis (PCA) and Pearson’s correlation coefficients were used to assess the global differences between the control and drought stress groups. For the drought, RNA-seq, the first principal component (PC1) explained 89.68%, while the second principal component explained 4.89%. Notably, the clear separation between the drought stress and control groups demonstrated that drought stress greatly influenced global gene expression profiles in both varieties (Figure 9a). PCA also showed that expression patterns for the same treatment within a specific variety were tightly grouped, whereas the distances between different treatments or varieties were significantly greater (Figure 9b).

#### 2.5.2. Transcriptomic Analysis of MS and HK Under Drought Stress

**Figure 10:**
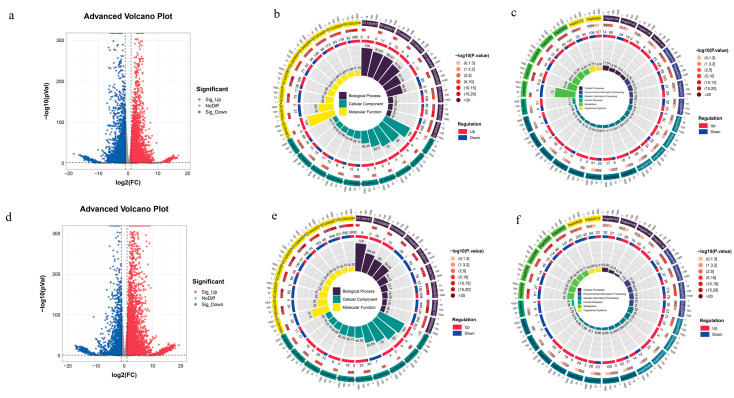
Transcriptomic Analysis of HK and MS under Drought Stress. (**a**) Differentially Expressed Genes in HK. (**b**) GO enrichment analysis of DEGs of HK_PEG vs. HK_CK group. (**c**) KEGG pathways of DEGs of HK_PEG vs. HK_CK group. (**d**) Differentially expressed genes in MS. (**e**) GO enrichment analysis of DEGs of MS_PEG vs. MS_CK group. (**f**) KEGG pathways of DEGs of MS_PEG vs. MS_CK group.

A total of 9577 differentially expressed genes (DEGs) were identified in the HK_PEG vs. HK_CK group, of which 6460 were found to be upregulated and 3117 downregulated (Figure 10a). In the group, a total of 6806 DEGs were annotated with GO functions, which were enriched in 1346 GO functions. These functions were classified into three categories: 589 in Biological Process, 133 in Cellular Component, and 624 in Molecular Function. The GO functions, based on *p*-values, include sequence-specific DNA binding (GO:0043565), polysaccharide binding (GO:0030247), and DNA-binding transcription factor activity (GO:0003700), among others (Figure 10b). A total of 3471 DEGs were annotated into 134 KEGG pathways. These 131 pathways were classified into six primary metabolic categories: 4 in Cellular Processes, 4 in Environmental Information Processing, 20 in Genetic Information Processing, 1 in Human Diseases, 103 in Metabolism, and 2 in Organismal Systems. KEGG pathways based on *p*-values are displayed, including arginine and proline metabolism (map00330), glyoxylate and dicarboxylate metabolism (map00630), starch and sucrose metabolism (map00500), among others (Figure 10c).

Figure 10d illustrates that the MS_PEG vs. MS_CK group contained a total of 8946 DEGs, with 4186 showing upregulation and 4760 exhibiting downregulation. In the group, a total of 6393 DEGs were annotated with GO functions, which were enriched in 1320 GO functions. These functions were classified into three categories: 573 in Biological Process, 139 in Cellular Component, and 608 in Molecular Function. The GO functions based on *p*-values, including protein kinase activity (GO:0004672), protein phosphorylation (GO:0006468), and glutathione transferase activity (GO:0004364), among others (Figure 10e). A total of 3147 DEGs were annotated into 135 KEGG pathways. These 135 pathways were classified into six primary metabolic categories: 4 in Cellular Processes,4 in Environmental Information Processing, 21 in Genetic Information Processing, 1 in Human Diseases, 103 in Metabolism, and 2 in Organismal Systems. KEGG pathways based on *p*-values were displayed, including starch and sucrose metabolism (map00500), glutathione metabolism (map00480), phenylpropanoid biosynthesis (map00940), among others (Figure 10f).

#### 2.5.3. Analysis of Transcriptional Regulation Mechanisms in DEGs Under Drought Stress

**Figure 11:**
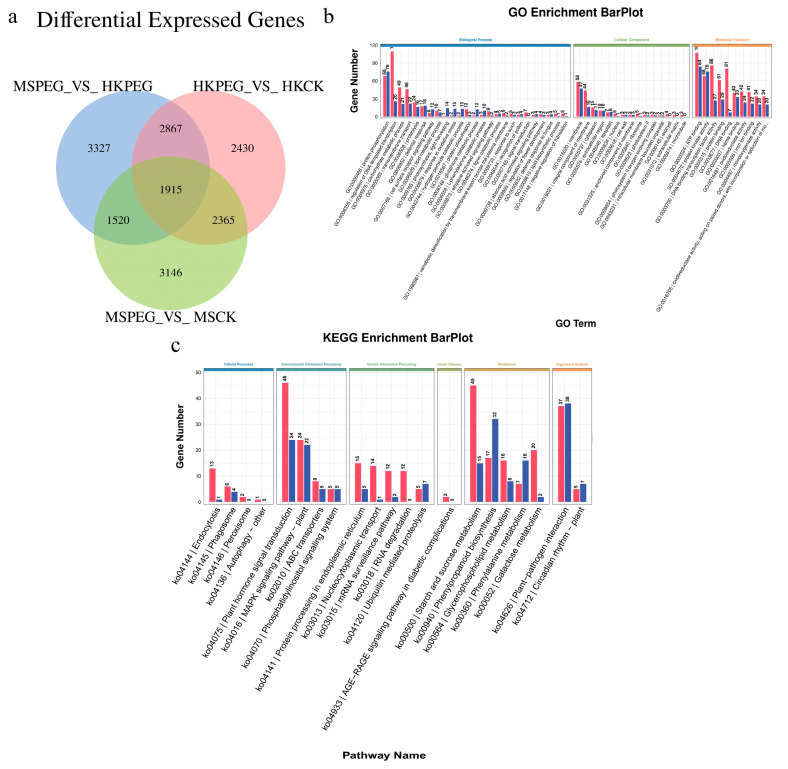
Differentially expressed genes (DEG) analysis under drought stress in oat. (**a**) Venn diagram illustrating the shared differentially expressed genes (DEGs) between HK-PEG vs. HK-CK, MS-PEG vs. MS-CK, and HK-PEG vs. MS-PEG. GO and KEGG enrichment analysis of overlapping 1915 DEGs. (**b**) GO enrichment (*p* < 0.05); (**c**) KEGG enrichment pathways (*p* < 0.05).

To enable a deeper investigation into the differences in drought stress responses among varieties, Venn diagrams were created for the different control groups (Figure 11). An overlap was observed in the differentially expressed gene sets, revealing 1915 common genes in the comparisons of HK-PEG vs. HK-CK, MS-PEG vs. MS-CK, and HK-PEG vs. MS-PEG. Accordingly, it was theorized that the 1915 genes with differential expression might be the main genes responding to drought stress in oats.

Figure 11b illustrates that out of the 1915 DEGs, 1368 were annotated with GO functions. These 1368 DEGs were enriched across 467 GO functions, which were classified into three categories: 193 in Biological Process, 33 in Cellular Component, and 241 in Molecular Function. The most prominent GO terms were associated with Molecular Function and Biological Process, with a significant number of DEGs involved in ATP binding (GO:0005524), protein kinase activity (GO:0004672), protein phosphorylation (GO:0006468), regulation of DNA-templated transcription (GO:0006355), and DNA-binding transcription factor activity (GO:0003700). Additionally, Figure 11c showed that 690 of the 1915 DEGs were annotated with KEGG functions. These 690 DEGs were mapped to 107 KEGG pathways, which are divided into six categories: 4 in Cellular Processes, 4 in Environmental Information Processing, 14 in Genetic Information Processing, 1 in Human Diseases, 82 in Metabolism, and 2 in Organismal Systems. Notably, the significantly enriched KEGG pathways included plant–pathogen interaction (ko04626), plant hormone signal transduction (ko04075), starch and sucrose metabolism (ko00500), phenylpropanoid biosynthesis (ko00940), and the MAPK signaling pathway.

#### 2.5.4. DEGs Involved in Plant Hormone Signal Transduction Pathways

**Figure 12:**
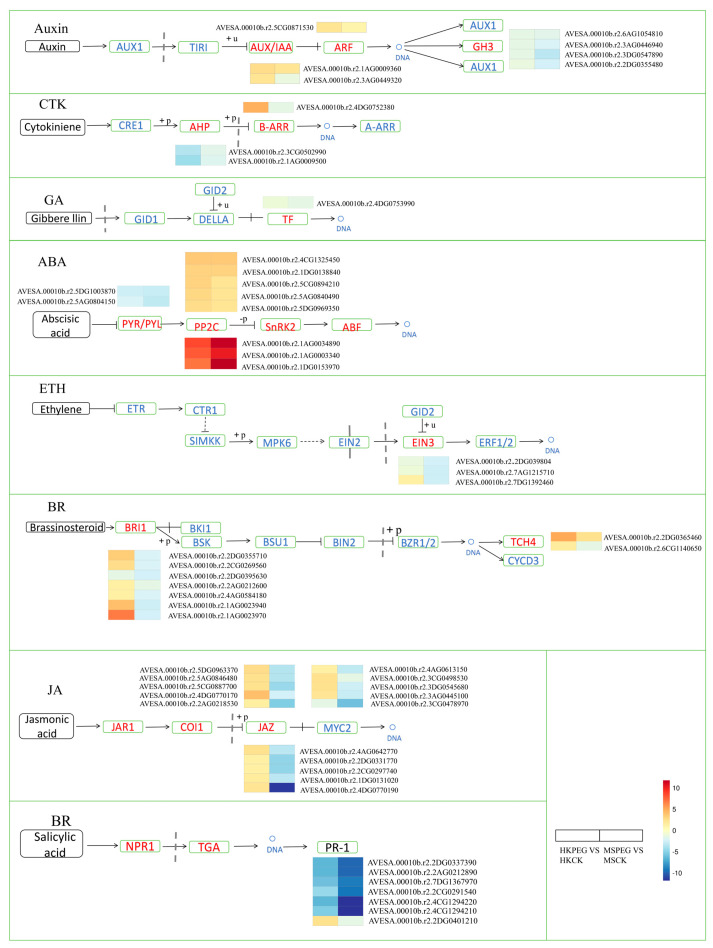
The plant hormone signal transduction pathway (ko04075) and the expression patterns of their associated DEGs are shown. The color scale ranges from min (blue) to max (red), indicating expression values from low to high.

It is widely acknowledged that plant hormone signaling pathways are vital for plant drought tolerance. Accordingly, KEGG enrichment analysis highlighted a significant enrichment of the plant hormone signal transduction pathways among the 1915 common DEGs. In order to better understand the influence of drought stress on plant hormone signal transduction-related genes in oats, a simplified pathway was constructed based on KEGG pathway analysis (Figure 12). The findings showed that 55 differentially expressed genes, which encode 14 enzymes involved in 8 hormone signaling pathways, were influenced by drought stress. In the ABA signaling pathway, drought stress led to a decrease in the expression of two abscisic acid receptor (*PYR*/*PYL*) genes in both varieties. Alternatively, eight *PP2C* genes experienced marked upregulation, with the MS variety showing a more significant increase than HK.

### 2.6. Weighted Gene Co-Expression Network Analysis

#### 2.6.1. WGCNA Module Correlation Analysis

**Figure 13:**
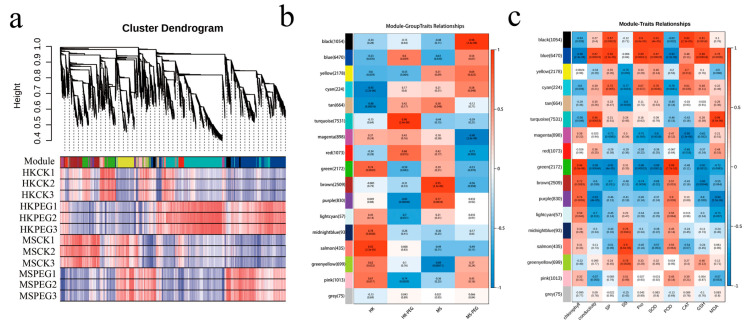
Identification and correlation analysis of WGCNA modules in oat under drought stress. (**a**) Hierarchical clustering tree of 17 co-expression modules identified using WGCNA. (**b**) Module–sample correlation. (**c**) Module–physiological correlation.

Weighted gene co-expression network analysis (WGCNA) is an important tool for identifying hub genes in RNA-seq analysis. In order to discover hub genes involved in the response to drought stress, we used the WGCNA R package (version 4.1.3 ) to cluster the expressed genes into different gene modules. In this WGCNA analysis, genes were grouped into 17 modules, with the turquoise module containing the highest number of genes (7531). The grey module was not analyzed in this study because it included all unclustered genes, making it of limited analytical importance (Figure 13a). As demonstrated in Figure 13b, the black module showed more pronounced changes in response to drought stress in the MS variety compared to the HK variety, whereas both varieties exhibited significant alterations in the blue module following drought treatment. Figure 13c showed the correlation between different modules and physiological indicators. The black, blue, cyan, and yellow-green modules showed a strong positive correlation with SP, PRO, SOD, CAT, GSH, and other indicators, while the pink and yellow modules were negatively correlated with conductivity and MDA. These six modules will be further analyzed.

#### 2.6.2. Hub Gene Screening

**Figure 14:**
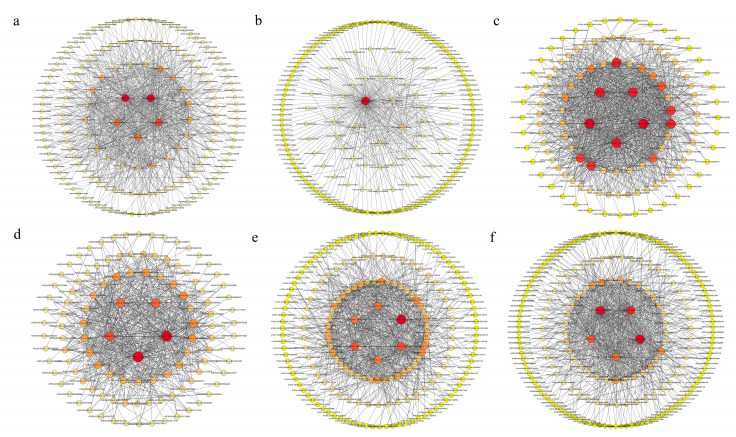
Hub genes in the candidate modules and their intramodular relationship. (**a**) Hub genes in black. (**b**) Hub genes in blue. (**c**) Hub genes in cyan. (**d**) Hub genes in green-yellow (**e**) Hub genes in pink. (**f**) Hub genes in yellow.

Cytoscape software (version 3.9.1) was employed to construct the transcriptional regulatory network. Figure 14a illustrates the network diagram of hub genes within the black module, emphasizing the top five genes identified as hub genes based on degree ranking: *AVESA.00010b.r2.5AG0827530* (*ARC5*), *AVESA.00010b.r2.2CG0275170* (*GDC1*), *AVESA.00010b.r2.5CG0916450* (*emb2726*), *AVESA.00010b.r2.6AG1024020* (*PPC2*), *AVESA.00010b.r2.6CG1135680* (*TYDC*).

Figure 14b depicts the network diagram of hub genes in the blue module, with the top five genes selected as hub genes according to degree ranking: *AVESA.00010b.r2.1AG0014000* (*PWD*), *AVESA.00010b.r2.1AG0020610* (*PAC*), *AVESA.00010b.r2.1AG0007940* (*PDE338*), *AVESA.00010b.r2.1AG0006060* (*EMB1865*), *AVESA.00010b.r2.1AG0030940* (*CIL*).

In Figure 14c, the network diagram of hub genes in the cyan module was presented, where the top five genes, determined by degree ranking, were identified as hub genes: *AVESA.00010b.r2.1AG0045940* (*CYP94C1*), *AVESA.00010b.r2.4AG0651620* (*PUMPKIN*), *AVESA.00010b.r2.5CG0931620* (*GSTZ2*), *AVESA.00010b.r2.6AG1010570* (*CCR1*), *AVESA.00010b.r2.1AG0003440* (*WTF1*).

Figure 14d also presented the network diagram of hub genes in the green-yellow module, highlighting the top five genes selected as hub genes based on degree ranking: *AVESA.00010b.r2.7CG0676360* (*ATHA2-1*), *AVESA.00010b.r2.5DG0949300* (*FAR1*), *AVESA.00010b.r2.2CG0306900* (*PGLP1*), *AVESA.00010b.r2.7AG1197660* (*FNR2*), *AVESA.00010b.r2.1AG0012500* (*CPGK2*).

Figure 14e showed the network diagram of hub genes in the pink module, where the top six genes, based on degree ranking, are identified as hub genes: *AVESA.00010b.r2.7DG1332750* (*FNR2*), *AVESA.00010b.r2.1AG0004780* (*FLA13*), *AVESA.00010b.r2.3CG0512960* (*FBP*), *AVESA.00010b.r2.1AG0026960* (*TPS14*), *AVESA.00010b.r2.2CG0291000* (*PLR1)*, *AVESA.00010b.r2.UnG1417910* (*ZKT*).

Figure 14f displayed the network diagram of hub genes in the yellow module, with the top five genes selected as hub genes according to degree ranking: *AVESA.00010b.r2.1CG0077990* (*RAB1B*), *AVESA.00010b.r2.1DG0179040* (*SCE1*), *AVESA.00010b.r2.1DG0174230* (*CRK29*), *AVESA.00010b.r2.2AG0262000* (*LRRAC1*), *AVESA.00010b.r2.2DG0356400* (*MUG3*).

#### 2.6.3. Correlation Analysis of HUB Genes

**Figure 15:**
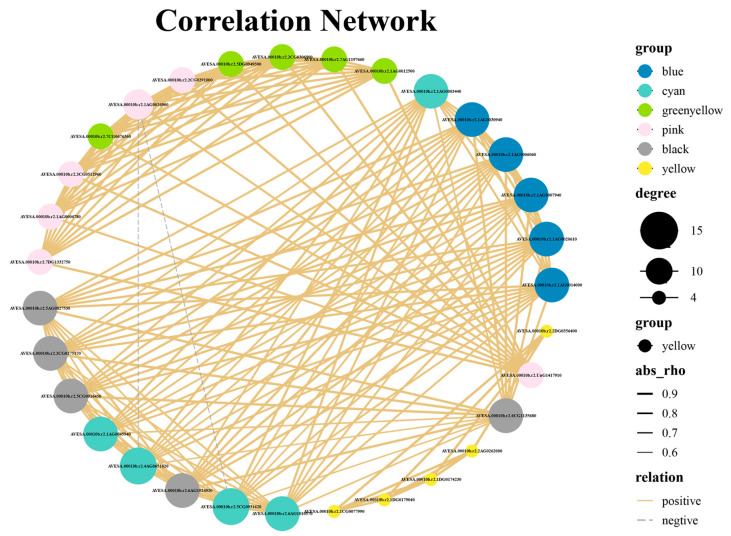
Co-expression network relationships of hub genes. The colors of the nodes indicate different groups, their sizes reflect connectivity levels, line thickness indicates absolute correlation coefficients, and solid or dashed lines distinguish between positive and negative correlations.

The study conducted a co-expression network analysis on 31 hub genes identified using WGCNA. Figure 15 shows the network diagram of co-expression among all core genes. The genes *AVESA.00010b.r2.4AG0651620* (*PUMPKIN*) and *AVESA.00010b.r2.5CG0931620* (*GSTZ2*) had the highest degree values, with 15 core genes showing co-expression relationships with them. Genes exhibiting higher co-expression connectivity were categorized into three distinct modules. The cyan module was composed of the following genes: *AVESA.00010b.r2.5CG0931620* (*GSTZ2*), *AVESA.00010b.r2.4AG0651620* (*PUMPKIN*), *AVESA.00010b.r2.6AG1010570* (*CCR1*) and *AVESA.00010b.r2.1AG0045940* (*CYP94C1*). The blue module encompassed the genes *AVESA.00010b.r2.1AG0030940* (*CIL*), *AVESA.00010b.r2.1AG0006060* (*EMB1865*), *AVESA.00010b.r2.1AG0007940* (*PDE338*), *AVESA.00010b.r2.1AG0020610* (*PAC*), and *AVESA.00010b.r2.1AG0014000* (*PWD*). The black module comprised the genes *AVESA.00010b.r2.6AG1024020* (*PPC2*), *AVESA.00010b.r2.5CG0916450* (*emb2726*), *AVESA.00010b.r2.2CG0275170* (*GDC1*), and *AVESA.00010b.r2.5AG0827530* (*ARC5*).

#### 2.6.4. Connection Between Physiological Indicators and Hub Genes

**Figure 16:**
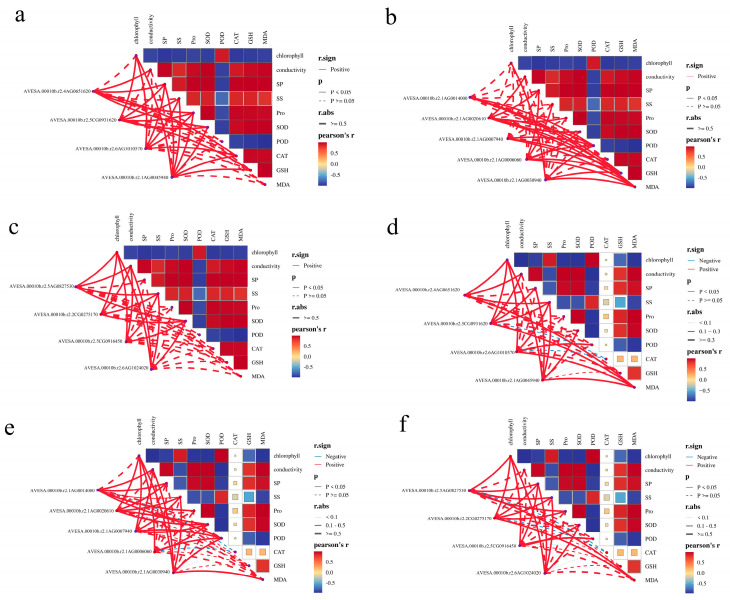
Relationship between hub genes responding to drought stress and physiological indicators in MS and HK. (**a**) Hub genes of cyan module in MS. (**b**) Hub genes of blue module in MS. (**c**) Hub genes of black module in MS. (**d**) Hub genes of cyan module in HK. (**e**) Hub genes of blue module in HK. (**f**) Hub genes of black module in MS. The red line represents positive correlation, and the blue line represents negative correlation. The x-axis represents the gene ID, and the y-axis represents the physiological index.

To investigate the correlation between hub genes and physiological indicators, a Mantel test analysis was conducted in this study (Figure 16). In the MS variety, all genes exhibited a positive correlation with all indicators. Conversely, in the HK variety, the genes *AVESA.00010b.r2.5CG0931620* (*GSTZ2*), *AVESA.00010b.r2.6AG1010570* (*CCR1*), *AVESA.00010b.r2.1AG0014000* (*PWD*), *AVESA.00010b.r2.1AG0006060* (*EMB1865*), *AVESA.00010b.r2.5AG0827530*(*ARC5*), and *AVESA.00010b.r2.2CG0275170* (*GDC1*) demonstrated a significant negative correlation with the CAT indicator.

### 2.7. RT-qPCR Validation

**Figure 17:**
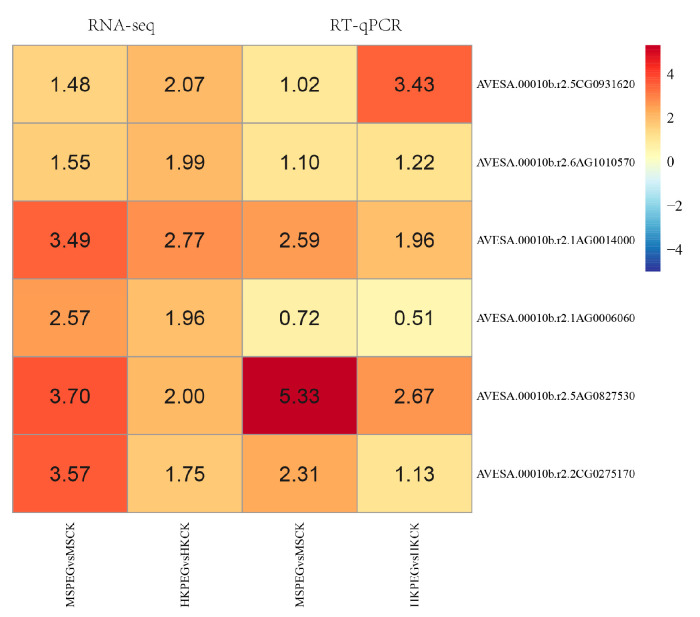
Validation of the RNA-seq data by real-time quantitative PCR (RT-qPCR) of six hub genes. The heatmap shows the log2 fold change values. For RT-qPCR analysis, each assay was performed with three biological replicates, and each biological replicate was analyzed with three technical replicates. *AsUBC* was used as the internal reference gene.

To validate the reliability of the RNA-seq data, this study conducted real-time quantitative PCR analysis on six selected hub genes. RT-qPCR validation showed that the expression patterns of the six candidate hub genes were consistent with the trends of the RNA-seq data, and significant expression differences were observed between varieties (Figure 17).

## 3. Discussion

### 3.1. Seed Germination Under Drought Stress Varies Among Different Oat Varieties

Seed germination, which initiates the life history of spermophytes, depends on water as a necessary condition. A lack of water will impact the function of internal enzymes, cell division, and various physiological metabolic processes in seeds. At the same time, insufficient water may result in seeds losing their vitality since they cannot absorb adequate water immediately, thereby impacting germination [17,18,19]. Thus, assessing the seed germination rate and growth indicators can be a crucial criterion for initially screening oat varieties for drought stress tolerance.

This study used K-means clustering to classify 28 oat varieties into four groups based on drought tolerance: drought-tolerant, moderately drought-tolerant, weakly drought-tolerant, and drought-sensitive. This analysis successfully identified drought-tolerant varieties (MS and MD) and drought-sensitive varieties (HK and HW).

### 3.2. Physiological Responses to Drought Stress Vary Between Drought-Tolerant and Sensitive Varieties

To further identify drought-tolerant and drought-sensitive varieties, physiological indicators were measured across the four identified categories. However, drought tolerance is a multifaceted trait affected by various factors, and using only one type of indicator for evaluation can result in mistakes [20,21]. At present, no single measure can fully and accurately assess drought tolerance. Therefore, it is crucial to adopt more comprehensive indicators and employ appropriate assessment methods to evaluate the drought resistance of oats [22].

Chlorophyll is a key component of photoprotection, directly influencing the efficiency of photosynthesis and serving as a signaling precursor for crop growth under drought stress [23]. It is reported that rice varieties resistant to drought retain more chlorophyll and carotenoids during drought conditions, demonstrating better performance [24]. Our experimental results were consistent with previous reports. At the beginning of drought stress, there was a significant reduction in chlorophyll content for HK and HW, while MS and MD’s levels stayed relatively constant. However, by the tenth day of drought stress, chlorophyll content had significantly decreased in all four varieties. This could be due to plants lowering their light capture efficiency to prevent additional harm to the photosynthetic system from reactive oxygen species buildup [25,26].

SS and SP are essential osmotic regulators involved in controlling osmotic pressure, maintaining protein and cell membrane stability, and eliminating excess free radicals [27,28,29]. Research on *Pinellia ternate* [30] and *Carex breviculmis* [31] showed a significant increase in SP content under moderate drought, while SS content decreased during both severe and moderate drought. Our results were consistent with these findings, showing that with prolonged drought stress, the SP content of all four varieties increased compared to the control. Except for MS, which showed a slight increase in SS content on the tenth day of stress, the SS content of the other varieties gradually decreased as the stress duration extended. The decrease in SS content might be due to the inhibition of SS synthesis, as drought stress causes a reduction in photosynthesis in the plants [32].

MDA [32] and REC [33] are both indicators that can show the level of damage to plant cell membranes due to drought stress and are crucial for evaluating plant drought tolerance. In this study, as drought stress continued, all four varieties showed an increase in MDA levels and REC.

Plants mainly counteract the harmful impacts of drought stress using intricate non-enzymatic and enzymatic antioxidant mechanisms. GSH levels play an essential role in the non-enzymatic antioxidant system and in maintaining cellular redox balance [34]. Meanwhile, enzymatic antioxidants consist of SOD, CAT, and POD [35,36]. SOD is a crucial antioxidant enzyme that is considered to play important roles in protecting the cells from cellular damage caused by ROS in living cells [37]. According to our study, the SOD content in all four varieties increased under drought stress. The duration of the drought extended, leading to a significant enhancement in SOD activity. POD and CAT are key antioxidant enzymes that help neutralize excess H_2_O_2_ by transforming it into water and oxygen, thereby averting harm to cells [38]. The study indicated that under drought stress, there was an increase in POD and CAT activities in all four varieties compared to the control, with CAT activity levels being particularly higher in MS and MD than in HK and HW. GSH acts as a non-enzymatic antioxidant by directly neutralizing ROS, which reduces oxidative harm [39]. This experiment showed that drought stress resulted in higher GSH content in all four varieties. The increase was comparable between drought-sensitive and drought-tolerant varieties, implying that GSH content changes were not a determining factor.

The applicability of grey relational analysis in the agricultural field has been extensively validated [40]. This study assessed the physiological indices of different oat varieties under drought stress using the grey relational analysis method and identified the drought-tolerant variety MS and the drought-sensitive variety HK.

### 3.3. Analysis of the Transcriptional Regulatory Mechanism of Oat Under Drought Stress

Since the drought resistance traits in plants are usually managed by multiple genes and the mechanisms are complex, further research is needed to explore the drought resistance mechanisms of different oat varieties at the molecular level. This study conducted a transcriptome analysis of both MS and HK varieties to clarify the molecular mechanisms responsible for their differing drought tolerance.

In response to drought stress, the MS and HK varieties reduced stress impacts by significantly adjusting the expression of specific genes, either increasing or decreasing their activity, relative to the control group [41]. The count of DEGs in HK during drought was much higher than in MS, possibly because drought-sensitive varieties required a broader activation of genes to cope with drought stress. To better compare the differences between the MS and HK varieties, we examined the overlapping DEGs shared by the three comparison groups: MS_PEG vs. MS_CK, HK_PEG vs. HK_CK, and MS_PEG vs. HK_PEG. This method successfully reduced biases caused by inherent differences among the varieties [42].

The KEGG analysis revealed that plant hormone signal transduction, starch and sucrose metabolism, and phenylpropanoid biosynthesis play roles in the oat’s response to drought stress. Plant hormone signaling is the core regulator of gene expression changes in response to abiotic stress. ABA plays a crucial role in the equilibrium of endogenous hormones and the regulation of growth metabolism [43]. The PYR/PYL receptors are positioned at the forefront of the negative regulatory pathway that modulates ABA signal transduction by inhibiting *PP2Cs* through ligand-induced interactions with *PP2C* [44]. Under drought stress treatment, both MS and HK significantly regulated *PP2C*, indicating the impact of drought stress on ABA signal transduction.

### 3.4. WGCNA-Based Exploration and Functional Analysis of Hub Genes

The WGCNA results in this study reveal that the black, blue, cyan, and yellow–green modules exhibited a significant positive correlation with drought-related traits, including SP, PRO, SOD, and other indicators. Conversely, the pink and yellow modules demonstrated a negative correlation with conductivity and MDA. These findings suggest that these gene modules may play a pivotal role in regulating drought resistance in oats.

We used Mantel test to analyze the correlation between the FPKM values of 13 core genes shared by two varieties and their physiological indicators. The results showed that in the drought-tolerant variety MS, all 13 core genes were positively correlated with the physiological indicator CAT, while in the drought-sensitive variety HK, 6 core genes were negatively correlated with CAT. This suggests that these 6 core genes are likely responsible for influencing the drought tolerance capabilities of the two oat varieties.

Among the six core genes, *AVESA.00010b.r2.5CG0931620* and *AVESA.00010b.r2.2CG0275170* have homologous genes in *Arabidopsis*, which are *AT2G02380* and *AT1G50900*. *AT2G02380* belongs to the glutathione S-transferase (GST) family. Previous studies have shown that GST family genes are involved in the drought stress response process in Chinese cabbage [45]. *AT1G50900*, as a core gene regulating chloroplast development, does not directly participate in drought stress signaling pathways. However, by maintaining the integrity of photosynthetic structures and regulating reactive oxygen species (ROS) homeostasis, it indirectly influences plant physiological adaptation mechanisms and post-stress recovery capacity during drought conditions [46].

## 4. Materials and Methods

### 4.1. Materials

Oat seeds were provided by the Forage Laboratory of Northeast Agricultural University. The variety and origin of the seeds are listed in Supplementary Table S3.

### 4.2. Design of Oat Seed Stress Tests

The full-grain oat seeds underwent a selection process to remove the palea and were subsequently rinsed with double-distilled water (ddH_2_O) to eliminate any residual chemicals. The concentration of PEG-6000 used for oat treatment in this study was determined to be 25%, based on preliminary germination tests. To ensure the reliability of the results, three replicate groups were established, with each group consisting of 30 disease-free seeds. The seeds were positioned in germination dishes with dimensions of 230 mm × 190 mm. Each dish was lined with three layers of filter paper. In the treatment group, the filter paper was pre-saturated with an equivalent volume of a 25% PEG-6000 solution, whereas in the control group, it was pre-saturated with an equivalent volume of ddH_2_O water. Throughout the cultivation period, the treatment group received a daily supplementation of the 25% PEG-6000 solution in equal volumes, while the control group was supplemented daily with an equivalent volume of ddH_2_O water. The dishes were maintained in a greenhouse environment with a photoperiod of 16 h of light per day, temperature settings of (25 ± 1) °C during the day and (18 ± 1) °C at night, and a relative humidity of 55%. To assess germination rate, germination energy, germination index, root length, and shoot length, three seedlings were randomly selected from each of the three independent replicate groups after 10 days of cultivation. A seed was considered germinated when the radicle emerged and extended to at least 2 mm, and a plant cultured for 10 days was defined as a seedling. Root and shoot lengths were measured directly using a straightedge. The germination rate, germination potential, and germination index, along with the relative values of each indicator, were calculated using the formula provided in Table 2. Subsequently, K-means cluster analysis was performed to identify two drought-tolerant varieties (MD, MS) and two drought-sensitive varieties (HK, HW) [47].

### 4.3. Design of Oat Seedling Stress Tests

The selected drought-tolerant and sensitive varieties underwent germination in Petri dishes for a duration of seven days. Subsequently, a total of 240 seedlings exhibiting nearly identical morphology were transferred to seedling boxes. Following the development of two true leaves, the seedlings were irrigated with Hoagland’s nutrient solution every two days. After a two-week acclimatization period, a stress treatment was initiated by incorporating 25% PEG-6000 into the nutrient solution, while the control group continued to receive only Hoagland’s nutrient solution. For the treatment group, the nutrient solution containing the 25% PEG-6000 was replenished every two days, and the control group received an equivalent volume of nutrient solution at the same intervals. Four distinct time points were established for each treatment: 3 days, 5 days, 7 days, and 10 days.

For each treatment group, two plants were randomly selected from each of the 3 biological replicates per treatment group. At each time point, the leaves and stems of the seedlings were harvested and immediately cryopreserved in liquid nitrogen. These samples were subsequently stored at −80 °C for future experimental analysis.

### 4.4. Analysis of Growth Indicators and Physiological Indices Under Drought Stress

Growth indicators included measurements of plant height using a straightedge and assessments of fresh and dry weights through a weighing method. Fresh weight was determined by directly measuring the above-ground portions of oat. Dry weight was obtained by placing the weighed fresh samples in an oven at 65 °C until a stable weight was achieved, followed by cooling and subsequent measurement of the dry weights.

The above-ground parts of the oat were subjected to grinding and homogenization for measurement purposes. The activities of the antioxidant system (SOD, POD, CAT) and the concentrations of various metabolites (GSH, MDA, proline, soluble protein, soluble sugar) in 1 mL of leaf sample supernatant were quantified according to the manufacturer’s instructions (Suzhou Keming Biological Co., Ltd., Suzhou, China). The specific formula for calculating relative water content was provided in Table 2. The REC of the blade was measured using a conductometer [48]. Chlorophyll content was measured through the anhydrous ethanol extraction method [49]. Subsequently, Grey correlation analysis, a multivariate statistical method that combines multiple trait indicators into a single correlation degree, was used to evaluate and rank drought tolerance among the subjects. The variety exhibiting the highest composite index was classified as drought-tolerant (MS), whereas the variety with the lowest composite index was deemed sensitive (HK).

**Table 2:** Correlation formulas between seed growth and physiological indicators.

Indices	Unit	Formula
germination rate (GR)	%	$\mathrm{GR}=\;\frac{Ne}{N}\times \;$100 [50] where N = the total number of seeds sown Ne = the number of seedlings that emerged.
Germination energy (GE)	%	$\mathrm{GE}=\;\frac{Gt}{Nt}$× 100 [51] where Gt = the average of the two days with the highest number of germinated seeds
germination index (GI)		$\mathrm{GI}=\frac{G1}{D1}$, $\frac{G2}{D2}$+ ⋯ $+\frac{Gn}{Dn}$ where Gn = number of seed newly germinated at time Dn; Dn = days from when set to germinate
*Cold* *tolerance* *indicator*		$Cold\;tolerance\;indicator=\frac{cold\;treatment\;group\;indicator}{control\;group\;indicator}$
relative water content	%	RWC(%) = [(FW − DW)/(TW − DW)] × 100 [52] FW is the fresh weight; Saturation weight (TW) was obtained by soaking the weighed oat leaves in water for 24 h and then removing them; DW is the dry weight.

### 4.5. RNA Extraction and Sequencing

To examine the transcriptional response of plants to drought stress, we conducted a transcriptome analysis utilizing the Illumina HiSeq 4000 platform. Following a 10-day period of drought stress and control treatment, the aerial parts of oats from the MS and HK varieties were collected, ground, and homogenized. For each variety, one cohort was subjected to control treatment while another cohort was exposed to drought stress, resulting in a total of four experimental groups. Each group was analyzed in three biological replicates. Total RNA was extracted from the samples using TRIzol reagent, and its quality was assessed via 1% denaturing agarose gel electrophoresis. RNA integrity was further evaluated using the Agilent 2100 Bioanalyzer system (Agilent Technologies, Santa Clara, CA, USA). mRNA was selectively enriched using oligo (dT) magnetic beads and subsequently fragmented using a fragmentation buffer. First-strand cDNA synthesis was performed using random hexamer primers, followed by second-strand synthesis employing M-MuLV Reverse Transcriptase (RNase H−), DNA Polymerase I, and dNTPs. The resulting cDNA was purified with AMPure XP beads. The double-stranded cDNA underwent end repair, adenylation, and adapter ligation. Fragments approximately 300 base pairs in length were then purified and amplified via PCR. The libraries were sequenced on the Illumina HiSeq 4000 platform, producing 150-base-pair paired-end reads.

### 4.6. Analysis of Data

Clean data, referred to as clean reads, were obtained by filtering out reads that contained adapters, reads with more than 10% ambiguous nucleotides (poly-N), and low-quality reads from the raw dataset. Simultaneously, the quality metrics Q20 and Q30, along with GC content and sequence duplication levels of the clean data, were calculated. The clean reads were then aligned to the reference genome (accessible at https://wheat.pw.usda.gov/GG3/content/avena-sang-download, accessed on 5 December 2023) using HISAT2. The initial transcript assembly for each sample was performed using StringTie (version 2.1.6, available at http://ccb.jhu.edu/software/stringtie/, accessed on 5 December 2023) with default parameters, and the mapped reads were subsequently merged to reconstruct a comprehensive transcriptome. The R package edgeR (version 4.1.3) was employed to identify statistically significant differences between samples, with DEGs defined by a fold change threshold of Log_2_FoldChange (LFC) of 2.0 and a *p*-value < 0.05. The differential gene set was further analyzed for GO functional enrichment and KEGG pathway enrichment using the GOseq (version 1.10.0) and KOBAS (version 2.0.12) software, respectively [53].

### 4.7. Validation of RNA-Seq by RT-qPCR

To validate the transcriptome data, nine genes were randomly selected for qRT-PCR analysis. The RNA utilized for qPCR was extracted from samples collected after ten days of drought treatment, ensuring consistency with the samples used in the transcriptomic analysis. Total RNA was extracted using an Ultrapure RNA Kit (CoWin Biotech, Beijing, China) in accordance with the manufacturer’s protocol. Complementary DNA (cDNA) synthesis was conducted using an oligo (dT) _10_ reverse primer, following the instructions provided with the HiScript II Q Select Reverse Transcriptase Kit (Vazyme Biotech, Nanjing, China). Primers were designed using Premier 5.0 software. Gene expression levels were quantified using the 2^−∆∆Ct^ method [54], with the *AsUBC* gene serving as the reference. The qPCR assays were performed using the 2× SYBR Green qPCR Master Mix II (Universal) (Seven Biotech, Beijing, China). The reaction procedure was as follows: 95 °C for 30 s, followed by 40 cycles of 95 °C for 5 s, 60 °C for 34 s. Subsequently, a melting curve analysis was performed from 60 °C to 95 °C at 0.3 °C increments to verify amplicon specificity [55].

### 4.8. WGCNA and Hub Gene Correlation Analysis

The gene co-expression network, developed through WGCNA, dynamically connects gene modules related to oat drought resistance with physiological markers [56]. The gene co-expression network was constructed utilizing the OmicStudio tools available at https://www.omicstudio.cn/tool, accessed on 1 March 2025. This analysis was predicated on the RNA-Seq data delineated in Section 4.5. The correlation matrix was developed by calculating the correlations between genes, which was subsequently transformed into a weight matrix to establish the gene co-expression network. The resulting analysis was visualized using Cytoscape software (version 3.9.1). Correlation analysis was conducted on the LC-Bio cloud platform (https://www.omicstudio.cn/tool, accessed on 10 March 2025) using the FPKM values of the hub genes in the conditions HK_CK, HK_PEG, MS_CK, and MS_PEG, with the outcomes visualized through the platform’s visualization tools. Furthermore, a Mantel test was conducted using the R package (version 4.1.3) to assess the relationship between FPKM values of genes related to drought stress and physiological indices across various varieties [57].

## 5. Conclusions

The study explored the mechanisms of drought resistance in oats by merging physiological and transcriptomic analyses of MS and HK varieties. MS’s outstanding adaptation to drought stems from its sustained osmotic regulation and redox balance, thereby mitigating oxidative damage and delaying chlorophyll degradation. Transcriptomic analysis revealed that the ABA signaling pathway is activated in response to drought stress. In the drought-tolerant variety MS, the overall upregulation of eight *PP2C* genes was higher than that in the sensitive variety HK, indicating that the differential regulation of the ABA signaling pathway is a key mechanism influencing drought resistance in oats. A co-expression network identified six central hub genes. By constructing a co-expression network that integrates physiological and transcriptomic data, core genes such as *AVESA.00010b.r2.5CG0931620* and *AVESA.00010b.r2.2CG0275170* were screened. Further research can focus on functional validation of these two central hub genes. In summary, this study provides valuable insights for further research on the effects of drought on the growth, development, and physiological characteristics of oats, offering new genetic resources for further investigation into oat drought responses.

## Figures and Tables

**Table 1:** Comprehensive evaluation of drought resistance of different varieties.

Membership Function Value	Group			
	MD	MS	HK	HW
Plant Height	0.996	0.99	0.957	0.893
Fresh Weight	0.987	0.992	0.998	0.952
Dry Weight	0.998	0.967	0.908	0.834
Chlorophyll	0.997	0.995	0.982	0.947
RWC	1	0.99	0.966	0.909
REC	0.996	0.998	0.972	0.931
MDA	0.993	0.964	0.968	0.965
PRO	0.815	0.937	0.566	0.333
GSH	0.982	0.975	0.973	0.801
SOD	0.998	0.974	0.905	0.79
POD	0.975	0.997	0.993	0.872
SP	0.943	0.964	0.851	0.797
SS	0.971	0.983	0.984	0.968
CAT	0.873	0.875	0.787	0.708
D	0.966	0.972	0.915	0.836
rank	2	1	3	4

## Data Availability

The RNA-seq datasets generated during the current study have been submitted to the NCBI Sequence Read Archive under the accession number: PRJNA1397469 (Release date: 2 February 2027). The raw data supporting the conclusions of this article will be made available by the authors on request.

## Supplementary Materials

The following supporting information can be downloaded at: https://www.mdpi.com/article/10.3390/plants15030453/s1. Table S1: Seed germination and growth indicators of 28 oat varieties under drought stress; Table S2: Statistical table for assessment of sample sequencing data; Table S3: 28 oat variety names and their origins.
